# Bariatric Surgery Reverses ORG and Exhibits a Distinct Transcriptomic Profile Compared to Weight Loss Through a Low-Fat Diet

**DOI:** 10.3390/ijms27020839

**Published:** 2026-01-14

**Authors:** Marina López-Martínez, Paula Rodríguez-Martínez, Lidia Blay, Pilar Armengol, Irina Pey, Mireia Ferrer, Esteban Porrini, Sergio Luis-Lima, Laura Díaz-Martín, Ana Elena Rodríguez-Rodríguez, Coriolano Cruz-Perera, Maruja Navarro-Díaz

**Affiliations:** 1 Department of Nephrology, Vall d’Hebron University Hospital, Pg. de la Vall d’Hebron, 119, 08035 Barcelona, Spain; marina.lopezmartinez@vallhebron.cat; 2 Department of Medicine, Universitat Autònoma de Barcelona, 08913 Barcelona, Spain; 3 Pathology Department, Germans Trias i Pujol Hospital, 08916 Badalona, Spain; prodriguez.germanstrias@gencat.cat; 4 General Surgery Department, Germans Trias i Pujol Hospital, 08916 Badalona, Spain; lydiablay@gmail.com; 5 Department of Surgery, Universitat Autònoma de Barcelona, 08913 Barcelona, Spain; 6 Genomic Platform, Germans Trias i Pujol’s Research Institute, 08916 Badalona, Spain; mparmengol@igtp.cat (P.A.); genomica@igtp.cat (I.P.); 7 Statistics and Bioinformatics Unit, Vall d’Hebron Research Institute, 08035 Barcelona, Spain; mireia.ferrer.vhir@gmail.com; 8 Laboratory of Renal Function (LFR), Faculty of Medicine, Complejo Hospitalario Universitario de Canarias, University of La Laguna, 38320 La Laguna, Spain; estebanporrini72@hotmail.com (E.P.); luis.lima.sergio@gmail.com (S.L.-L.); lauradiazmart@gmail.com (L.D.-M.); kokokruz@hotmail.com (C.C.-P.); 9 Instituto de Tecnologías Biomédicas (ITB), Faculty of Medicine, University of La Laguna, 38320 La Laguna, Spain; 10 Department of Laboratory Medicine, Complejo Hospitalario Universitario de Canarias, 38320 La Laguna, Spain; 11 Research Unit, Hospital Universitario de Canarias, 38320 La Laguna, Spain; anarrguez@gmail.com; 12 Fundación General de la Universidad, University of La Laguna, 38320 La Laguna, Spain; 13 Nephrology Department, Sant Joan Despí Moisès Broggi Hospital, c/d’Oriol Martorell, 12, 08970 Sant Joan Despí, Spain

**Keywords:** obesity-related glomerulopathy, bariatric surgery, low-fat diet, obesity glomerulopathy reversion, transcriptomic changes, Wistar rats

## Abstract

Weight loss is central to treating obesity-related kidney disease, yet the renal effects of a low-fat diet (LFD) versus bariatric surgery (BS) remain incompletely understood. This study compared their impact on obesity-related glomerulopathy (ORG). Twenty-eight male Wistar rats were fed a high-fat diet (HFD) for 10 weeks to induce obesity. Eight rats were sacrificed (the HFD group), eight switched to LFD for 10 weeks, and twelve underwent sleeve gastrectomy. Body weight, albuminuria, renal histology, and transcriptomic profiles were analyzed. Weight loss was modest in the LFD group (−1.6%) but substantial after BS (−13.2%), occurring 2.1 times faster. Albuminuria decreased in both interventions compared to HFD (LFD: 7228 ± 514 ng/mL; BS: 6242 ± 418 ng/mL; HFD: 10,384 ± 1168 ng/mL; *p* < 0.01) and correlated strongly with weight loss (R^2^ = 0.78). The glomerular area was reduced in both groups, but only BS achieved complete histological resolution of ORG. Tubular cells in BS-treated rats showed megamitochondria and cristae disruption, while LFD induced milder alterations. Transcriptomics revealed suppression of mitochondrial maintenance genes and upregulation of oxidative stress and immunometabolic pathways. Immune-related genes upregulated in BS clustered into pro-inflammatory/chemotactic and regulatory modules. To the best of our knowledge, this is the first piece of evidence that BS fully reverses ORG, highlighting renal effects beyond weight loss alone.

## 1. Introduction

The rising prevalence of obesity has brought with it a range of comorbidities, including the silent threat of chronic kidney disease (CKD) [1,2]. Obesity-related glomerulopathy (ORG) has emerged as the early stage of CKD in patients with obesity [3,4]. This subtype of nephropathy is characterized by hyperfiltration and proteinuria [5,6,7]. While hyperfiltration predisposes individuals to CKD, proteinuria is considered an initial stage of CKD and a recognized cardiovascular risk factor that exponentially increases mortality risk [8]. Unlike advanced CKD, ORG is potentially reversible, providing an opportunity for therapeutic intervention [9,10]. Therefore, improving our understanding of ORG development and implementing prompt, effective therapeutic measures could mitigate cardiovascular risk and mortality.

Maladaptive cellular and hormonal responses, abnormal lipid metabolism, and systemic chronic inflammation appear to play an important role in ORG development [3,4]. However, the pathways through which obesity might cause renal disease are not fully understood, since not all obese individuals develop obesity-related CKD. Our working group has recently developed a murine model of ORG. We confirmed that obese rats fed with a high-fat diet (HFD) had a differential RNA/miRNA signature in both kidney tissue and urine [11]. The integration of pathway-level information and consensus graph strategies can enhance biological interpretability, robustness, and functional insight, and places transcriptomic findings within the broader context of network-based analysis frameworks [12]. These findings contribute to our understanding of the biological mechanisms involved in the early stages of ORG, highlighting potential non-invasive biomarkers and therapeutic targets for ORG [13,14].

While there is still no specific target for ORG treatment, weight loss has long been the primary therapeutic option. The so-called “clinically relevant weight loss”, typically defined as a weight loss of ≥5–10%, results in significant improvements in cardiometabolic risk factors associated with obesity [15]. A low-fat diet (LFD) alone has rarely been able to achieve such drastic weight loss or maintain it in the long term [16,17]. Certainly, bariatric surgery has been, for many years, the only therapeutic option capable of achieving and maintaining clinically relevant weight loss over time. Furthermore, although other methods of drastic weight loss are effective, they rely heavily on long-term adherence to therapy to sustain their benefits [18,19].

From a renal perspective, bariatric surgery has clear long-term benefits. Most available evidence on the renal effects of bariatric surgery in obesity-related kidney disease is derived from clinical observational studies and experimental models focusing on functional outcomes, such as albuminuria, hyperfiltration, or inflammatory markers [9,10,16,19,20,21,22,23,24,25,26,27,28]. Although these studies consistently demonstrate improvements in renal function and proteinuria after bariatric surgery, they do not address whether these changes are accompanied by true structural and molecular reversal of obesity-related glomerulopathy. In particular, data regarding histological reversibility and transcriptomic remodelling of the kidney following bariatric surgery are currently lacking [28,29,30]. Our working group observed that patients with a kidney biopsy diagnosis of ORG could improve or even reverse proteinuria and hyperfiltration after bariatric surgery [9]. Moreover, other studies have shown that patients with severe obesity and CKD also experienced amelioration in inflammation biomarkers after bariatric surgery [10,28]. However, the histological effects of bariatric surgery on the kidneys and its impact on the gene expression of ORG have never been evaluated.

All in all, comparing the effects of two therapeutic approaches for treating obesity, such as bariatric surgery and LFD, will provide valuable insights into their differential impact on ORG pathophysiology. To date, no study has systematically compared bariatric surgery with dietary weight loss in an experimental model of ORG while integrating functional, histological, and transcriptomic analyses. Furthermore, whether bariatric surgery induces a complete reversal of ORG at the molecular level, rather than partial functional improvement, remains unknown. By incorporating kidney histology together with bulk transcriptomic profiling, the present study extends previous work by providing mechanistic insight into the biological pathways underlying ORG reversibility. Our goal is to determine whether bariatric surgery can achieve a complete functional, histological, and transcriptomic reversal of ORG, a possibility that has not yet been demonstrated in experimental models.

## 2. Results

### 2.1. Effects of Low-Fat Diet and Bariatric Surgery on Body Weight and Albuminuria in Wistar Rats

**Figure 1:**
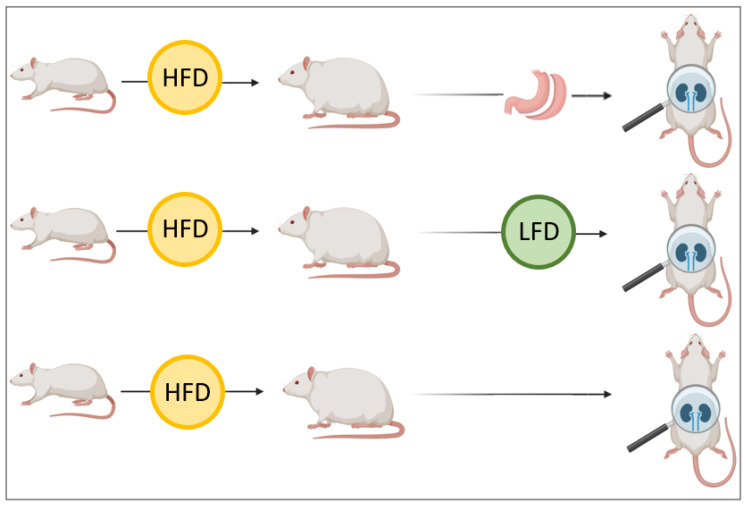
Experimental design and study groups. Twenty-eight rats were fed a high-fat diet (HFD) for 10 weeks to induce obesity. Thereafter, 8 rats were sacrificed (the HFD group), 8 were switched to a low-fat diet (the LFD group) for 10 weeks, and 9 underwent sleeve gastrectomy (the BS group). All groups were evaluated for body weight, albuminuria, kidney histology, and transcriptomic profiles. Created in BioRender.

An overview of the experimental design, including the allocation of animals to the different study groups, is shown in Figure 1. The effects of a low-fat diet (LFD) and bariatric surgery (BS) on weight loss were analyzed in Wistar rats. Rats in the LFD group exhibited an initial body weight of 344.32 ± 10.50 g, which increased to 483.83 ± 28.18 g before starting the dietary intervention, reflecting a 40.5% weight gain from baseline. After 10 weeks on the LFD, the animals experienced a slight weight reduction of −1.58%, reaching a final weight of 476.16 ± 26.06 g. The weight loss plateaued, and no further significant modifications were observed until the end of the study. The BS group had a baseline weight of 345.34 ± 6.54 g, which increased to 502.08 ± 21.99 before surgery, corresponding to a 45.38% weight gain. Six weeks post-surgery, the rats exhibited a significant weight loss of −13.2%, maintaining this trend despite being fed a standard diet. The rate of weight loss, calculated as the change in relative weight per week, was 2.1 times higher in the BS group compared to the LFD group, indicating a more rapid and sustained reduction in body weight following surgical intervention.

**Figure 2:**
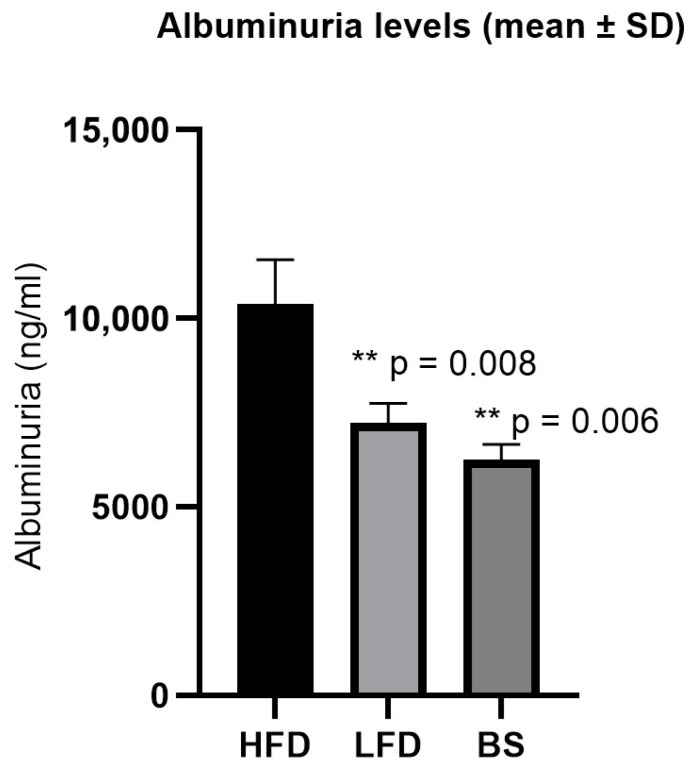
Albuminuria levels across experimental groups. Albuminuria values are expressed as mean ± standard deviation (SD). Rats in the HFD group showed significantly higher albuminuria compared with both intervention groups. Albuminuria levels were significantly reduced in the LFD group (*p* = 0.008) and in the BS group (*p* = 0.006) when compared with HFD. HFD: high-fat diet; LFD: low-fat diet; BS: bariatric surgery.

Albuminuria showed a clear decrease in both treatment groups when compared with the HFD (10,384.04 ± 1167.96 ng/mL) (Figure 2). In the LFD group, the levels reached 7228.04 ± 514.22 ng/mL (*p* = 0.008). In the BS group, albuminuria was 6241.50 ± 418.33 ng/mL (*p* = 0.006). Overall, the reduction in albuminuria was consistent across treatments. A strong association was also observed between albuminuria and weight loss (R^2^ = 0.78), indicating that lower albuminuria values were accompanied by greater body weight reductions. To further evaluate differences among experimental groups, albuminuria data were analyzed using a one-way ANOVA including HFD, LFD, and BS groups. This analysis revealed a significant group effect on albuminuria levels (*p* = 0.0083). Post hoc comparisons confirmed that both intervention groups showed significantly reduced albuminuria compared with the HFD group.

### 2.2. Study of Renal Morphological Changes in LFD and BS Rats

**Figure 3:**
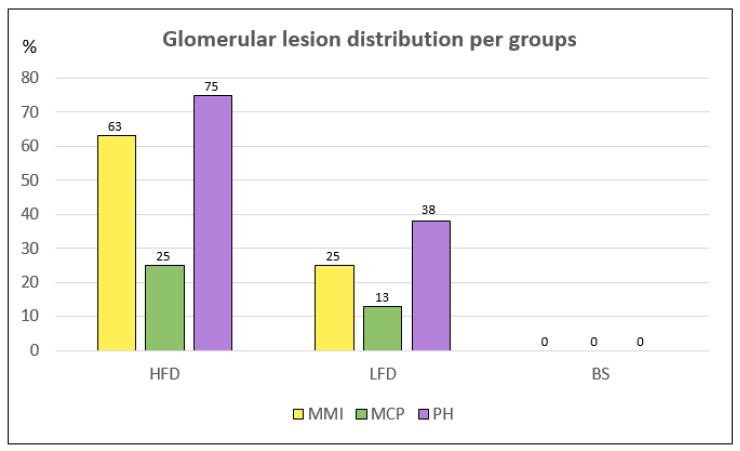
Glomerular lesion distribution per group. Rats fed with a high-fat diet (HFD) were the ones with the highest percentage of mesangial matrix increase (MMI), mesangial cell proliferation (MCP), and podocyte hypertrophy (PH), as well as the highest glomerular area. In the group of rats treated with the low-fat diet (LFD), glomerular lesions persisted but were reduced in number. In contrast, the bariatric surgery group (BS) showed no signs of obesity-related glomerulopathy and exhibited the smallest glomerular area.

**Figure 4:**
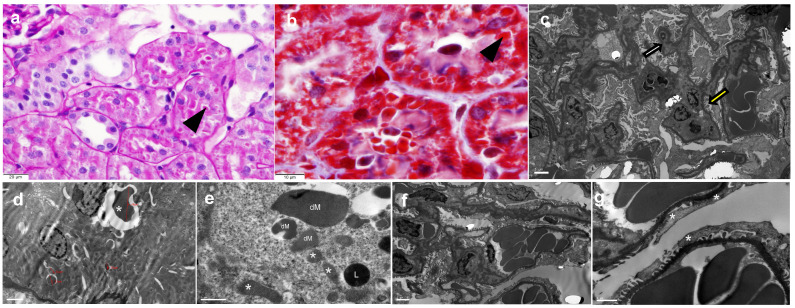
Identification of mitochondrial inclusions and podocyte alterations in kidneys following dietary and surgical interventions. (**a**) The BS group showing preserved tubular architecture with cuboidal epithelial cells, eosinophilic cytoplasm, centrally located nuclei, and the presence of round, brightly eosinophilic cytoplasmic inclusions (black arrow) (PAS stain, original magnification of ×400). (**b**) Masson’s trichrome staining of the BS group highlights cytoplasmic granularity in proximal tubular epithelial cells and a fuchsinophilic appearance of intracytoplasmic inclusions (black arrow) (original magnification of ×1000). (**c**) Ultrastructural analysis revealed normal glomeruli in the BS group, with no evident podocyte alterations. Podocyte foot processes (yellow arrow) were well defined, maintaining intact slit diaphragms. The glomerular basement membrane (white arrow) exhibited apparent thinning due to paraffin processing artefacts (TEM, original magnification of ×5000). (**d**) The intracytoplasmic inclusions observed on light microscopy correspond to megamitochondria (*) and dysmorphic mitochondria with loss of cristae structure, measuring 0.9–4.5 µm (TEM, ×8000). (**e**) Electron microscopy of the BS group showing proximal tubules containing membrane-bound inclusions identified as megamitochondria and dysmorphic mitochondria. Some mitochondria preserved normal cristae patterns (*), while others displayed disrupted cristae, increased size, and heterogeneous morphology (dM). Lysosomes (L) were also observed (TEM, original magnification of ×25,000). (**f**,**g**) Glomeruli from the LFD group were normocellular, with focal podocyte foot process effacement involving approximately 10% of the examined capillary loops (*). The glomerular basement membrane exhibited apparent thinning due to processing artefacts (TEM, original magnifications of ×6000 and ×20,000, respectively). Histological features of obesity-related glomerulopathy in HFD and LFD groups have been previously reported [11] and are therefore not shown here.

The kidneys of all groups were encased in a thick to moderate adipose tissue layer, and macroscopically, the kidneys from the HFD group looked larger. The HFD group showed typical histological lesions of ORG (Figure 3). The LFD group also exhibited the typical ORG lesions, although to a lesser extent (Figure 3). In contrast, none of the rats in the BS group showed any ORG lesions, but tubules showed some images resembling mitochondria (Figure 3 and Figure 4a,b). Because glomerular lesions were assessed as presence/absence, data were analyzed as frequencies rather than continuous variables. Notably, no glomerular lesions were observed in the BS group.

**Figure 5:**
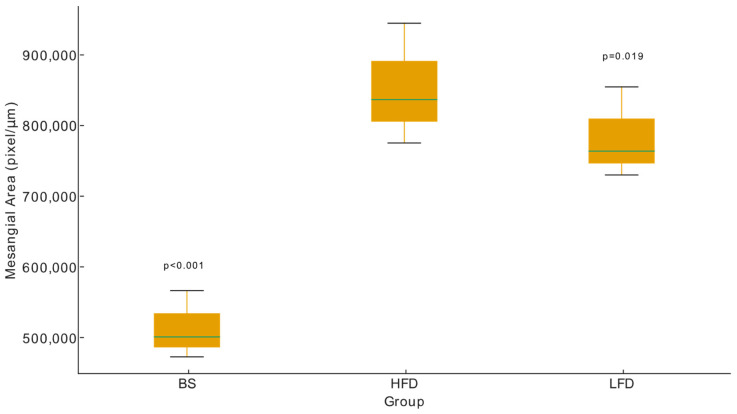
Distribution of mesangial area in BS, HFD, and LFD experimental groups.

Morphometric analysis revealed a higher MMI (pixels/µm) in the LFD group than in the BS group (Figure 5). Compared with the HFD glomerular area (10,640.92 ± 1220.62 µm^2^), the LFD group and BS group had glomerular areas that were significantly smaller (9109.76 ± 1185.51 µm^2^ and 8218.31 ± 916.72 µm^2^, *p* = 0.019 and *p* < 0.001, respectively). A significant group effect was observed for glomerular morphometric parameters via a one-way ANOVA. Electron microscopy demonstrated a complete resolution of glomerular alterations in the BS group, as glomeruli and podocytes appeared normal with no evidence of podocyte foot process effacement (Figure 4c). In contrast, the LFD group still exhibited minimal residual glomerular damage, with podocyte foot process effacement affecting approximately 10% of capillary loops (Figure 4f,g). Regarding tubular mitochondria, the BS group displayed numerous megamitochondria and dysmorphic mitochondria in proximal tubules, indicating persistent mitochondrial abnormalities (Figure 4d,e).

### 2.3. Treatment-Induced Transcriptomic Changes in Rat Kidneys

In order to identify the profile of genes that showed treatment-related changes in the kidney, we compared the level of gene expression using RNA-Seq experiments in HFD (*n* = 8), LFD (*n* = 8), and rats subjected to bariatric surgery (BS, *n* = 9) using three approaches: (1) comparison of LFD vs. HFD; (2) BS vs. HFD; and (3) BS vs. LFD. A total of 13,700 sequences were detected, and hierarchical clustering and principal component analysis were used to investigate the potential identification of groups, based on their molecular expression profile.

#### 2.3.1. Kidney Transcriptomic Changes in the Comparison of LFD vs. HFD Groups

The analysis and the heatmap revealed a tendency to mix both groups, but not completely (Supplementary Figure S1a). LFD and HFD samples clustered closely, indicating that these samples have a similar RNA signature. We labelled genes that were differentially expressed as those with an adj *p*-value < 0.02 with a log_2_FoldChange > 1. Following this criterion, the number of genes clearly differentially expressed was 30, and all of them were overexpressed in LFD rats (Supplementary Figure S1b–c). There were three underexpressed genes in LFD rats with an adj *p*-value < 0.03 with a log_2_FoldChange > 0.5 (Supplementary Figure S1c). The global analysis of Biological Significance was applied according to the Gene Ontology categories and Reactome Pathway analysis (Supplementary Figures S1e and S2). The biological processes include mRNA processing, mRNA splicing via the spliceosome, and RNA splicing via transesterification reactions as well as via transesterification reactions with bulged adenosine as nucleophiles (Supplementary Figure S2a). The cellular components mainly involved were integral and intrinsic components of the synaptic membrane, nuclear speck, respiratory chain, and spliceosomal complex (Supplementary Figure S2b). The molecular functions were chromatin binding, extracellular ligand-gated ion channel activity, histone binding, mRNA binding, and postsynaptic neurotransmitter receptor activity (Supplementary Figure S2c). The Reactome analysis showed that the enriched pathways are related to RNA metabolism, mRNA splicing, collagen degradation, and immune-regulatory interactions between a lymphoid and a non-lymphoid cell, among others (Supplementary Figure S1e).

#### 2.3.2. Kidney Transcriptomic Changes in the Comparison of BS vs. HFD Groups

The analysis and the heatmap revealed a tendency to mix both groups (Supplementary Figure S3a). BS and HFD samples clustered closely, but with the BS group showing a particular RNA signature (Supplementary Figure S3b). We labelled genes that were differentially expressed as those with an adj *p*-value < 0.01 with a log_2_FoldChange > 1. Following this criterion, the number of genes that were clearly differentially expressed was 143, and all of them were overexpressed in BS. We also found 11 genes that were increased in HFD that met the criteria of an adj *p*-value < 0.01 with a log_2_FoldChange < 1 (Supplementary Figure S3b). The first 20 main genes that are differentially expressed are given in Supplementary Figure S3c. The global analysis of Biological Significance was applied according to the Gene Ontology categories and Reactome Pathway analysis (Supplementary Figures S3 and S4). The biological processes include activation of immune response, adaptative immune response, leukocyte differentiation, negative regulation of immune system processes, and regulation of leukocyte activation (Supplementary Figure S4a). The cellular components mainly involved were the external side of the plasma membrane, the extracellular matrix, the membrane raft, the receptor complex, and the side of the membrane (Supplementary Figure S4b). The molecular functions were calcium ion binding, endopeptidase activity, receptor–ligand activity, receptor–regulator activity, and transmembrane signalling receptor activity (Supplementary Figure S4c). The Reactome analysis showed that the enriched pathways are related to immune system activation, lymphocyte differentiation, and regulation of immune responses, among others (Supplementary Figure S3e).

#### 2.3.3. Kidney Transcriptomic Changes in the Comparison of BS vs. LFD Groups

**Figure 6:**
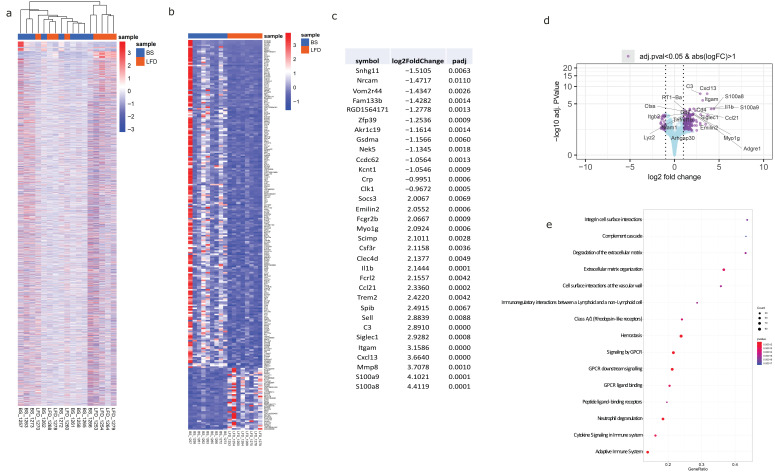
Kidney transcriptomic changes in the comparison of BS vs. LFD groups. (**a**) Heatmap comparing the expression profiles of all sequenced genes among BS and LFD rats. It should be noted that while some mixing of BS and LFD clusters is observed at this global level, indicating shared physiological adaptations to weight loss, more specific differential gene expression and pathway analyses (**b**–**e**) reveal distinct molecular signatures for each intervention. (**b**) Heatmap and table (**c**) of DE-selected genes for subsequent studies. (**d**) Volcano plot. This plot depicts, on the abscissa axis, the “biological change” (shown with the log fold change), and on the ordinate axis, the “statistical significance” (shown with the adjusted *p*-value). Genes are shown in purple when the adjusted p-value is under 0.05 and the absolute logarithmic fold change is above 1. Gene symbols are shown for the top 20 most significant genes. (**e**) Dot plot for the top 15 enriched Reactome pathways for comparison of BS vs. LFD groups.

**Figure 7:**
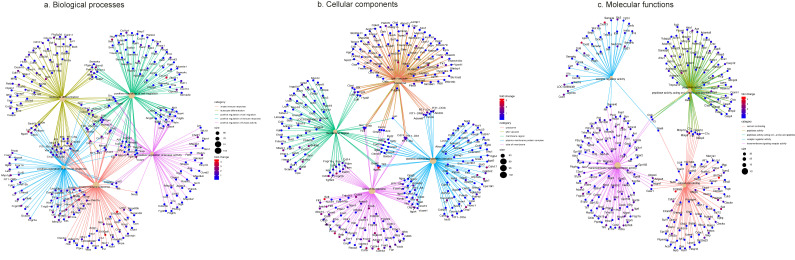
Gene Ontology (GO) analysis. Network plot for the 5 top GO-biological process (**a**), GO-cellular component (**b**), and GO-molecular function categories for the comparison of BS vs. LFD groups (**c**).

The analysis and the heatmap revealed a tendency to mix both groups (Figure 6a). The volcano plot showed that most genes were expressed at a similar level in BS and LFD, but there was one group of genes that was mostly present in BS and another group that was more highly expressed in LFD (Figure 6d). We considered genes to be differentially expressed based on two criteria: for overexpressed genes, an adjusted *p*-value < 0.01 and log_2_FoldChange > 1; for underexpressed genes, an adjusted *p*-value < 0.02 and log_2_FoldChange < −1. According to these thresholds, 105 genes were identified as overexpressed in BS rats, while 16 genes were significantly underexpressed. (Figure 6b). The first 33 differentially expressed genes are given in Figure 6d. The global analysis of Biological Significance was applied according to the Gene Ontology categories and Reactome Pathway analysis (Figure 6e). The biological processes include innate immune response, leukocyte differentiation, positive regulation of cell migration, positive regulation of immune response, and positive regulation of kinase activity (Figure 7a). The cellular components mainly involved were lysosomes, lytic vacuoles, membrane regions, the plasma membrane protein complex, and the side of the membrane (Figure 7b). The molecular functions were calcium ion binding, peptidase activity acting on L-amino acid peptidase, receptor–regulator activity, and transmembrane signalling receptor activity (Figure 7c).

To analyze whether the group that underwent bariatric surgery exhibits differential gene expression, we compared gene expression in the BS vs. LFD analysis and the BS vs. HFD analysis. Supplementary Table S1 presents the genes that are exclusively and differentially overexpressed in the BS group.

## 3. Discussion

To our knowledge, this is the first study to demonstrate the complete reversal of obesity-related glomerulopathy (ORG) after bariatric surgery (BS) alongside a profound remodelling of renal tubular mitochondria. Both the low-fat diet (LFD) and BS groups achieved weight loss and a reduction in albuminuria (Figure 2). A key theoretical distinction emerged: only the BS group achieved complete histological resolution of ORG (Figure 3), whereas LFD led to only partial improvement. This differential outcome is likely driven by the unique interplay of several factors induced by BS that extend beyond simple caloric restriction, including the magnitude and rapidity of weight loss, a distinct pattern of mitochondrial remodelling, and a specific renal immunometabolic signature, although only the BS group resolved all glomerular lesions of ORG (Figure 3). While previous work [29,31] has reported improvements in glomerular morphology and mitochondrial function in extra-renal tissues following bariatric procedures, our study is the first to demonstrate the coexistence of restored glomerular morphology and mitochondrial function in the kidney (Figure 4). Moreover, transcriptomic analysis further revealed a differential mRNA profile in the BS group in comparison with LFD and high-fat-diet (HFD) groups (Supplementary Table S1), supporting the notion that BS profoundly impacts renal gene expression through these multi-faceted mechanisms. This pattern suggests that BS has profound impacts on renal gene expression, potentially through mechanisms beyond simple caloric restriction.

Dietary treatments for obesity are well known to be difficult to maintain, often resulting in weight gain after an initial rapid loss [18,19,21]. The specifics of our sleeve gastrectomy technique, notably the consistent 70% gastric resection, are critical factors influencing the observed profound outcomes. This precise amount of stomach removal, achieved through a method adapted for Wistar rats from human surgical principles, ensures a substantial and sustained caloric restriction while preserving the pyloric sphincter and vagal integrity. This specific quantity of resection is crucial as it directly dictates the magnitude of anatomical and physiological alterations, impacting hormonal signalling, gut microbiota changes, and nutrient absorption, which collectively contribute to the profound metabolic shifts observed. The significant and rapid weight loss observed in our BS group is a direct consequence of this intervention, distinguishing its physiological impact from the gradual weight reduction seen with LFD. While the procedure’s efficacy in achieving ORG reversal was robust, the potential for minor inter-animal variability in the precise extent of resection is an inherent aspect of rodent surgical models, though our consistent results across the group suggest high technical proficiency. This precise control over the surgical parameter allows for a strong interpretation of the distinct metabolic and renal responses observed, including the unique mitochondrial remodelling and immunometabolic signatures, as direct consequences of the profound physiological changes induced by this specific bariatric intervention. Indeed, the LFD group experienced an initial weight loss that then stabilized, whereas the BS group continued to lose weight throughout the follow-up period. These results suggest that while the LFD led to stabilization of weight with minimal reduction, the BS resulted in a more pronounced and sustained weight loss [16,21]. Interestingly, the reduction in albuminuria in both treatment groups correlated with weight loss (Figure 2). The reduction in albuminuria following bariatric surgery has previously been demonstrated, although most studies have included mixed populations of patients with both obesity and type 2 diabetes [10,20,22,23,25,26,27,29,32,33,34].

This study provides new evidence on the renal effects of obesity treatment in Wistar rats, with particular emphasis on mitochondrial remodelling. As we have previously reported, diet-induced obesity in Wistar rats is accompanied by the development of ORG [11], which served as the baseline renal phenotype in this study. Following treatment, the two interventions diverged markedly. While LFD-treated rats still retained at least one histological feature of ORG and continued to show podocyte effacement with only minor evidence of mitochondrial remodelling (Figure 3 and Figure 4f,g), BS-treated rats exhibited complete resolution of glomerular lesions and normalization of podocyte architecture (Figure 3 and Figure 4c). Bariatric surgery has previously been associated with improved mitochondrial function [28] and reductions in renal inflammation, fibrosis, and glomerular structure [29,30]. Consistently, the BS group showed tubular cells with abundant inclusions that stained red with Masson’s trichrome (Figure 4b). Ultrastructural analysis revealed that these inclusions corresponded to megamitochondria and dysmorphic mitochondria with disrupted cristae, indicating a profound mitochondrial remodelling process (Figure 4d,e). Remarkably, this is the first study to demonstrate a complete histological reversal of ORG following bariatric surgery.

The presence of dysmorphic mitochondria in renal tubular cells following bariatric surgery merits further consideration. One plausible explanation for this phenomenon is that the rapid weight loss and subsequent metabolic shifts following bariatric surgery place significant energy demands on renal tubular cells. This increased metabolic burden, coupled with potential ischemia–reperfusion injury associated with the surgical procedure, might initially overwhelm mitochondrial capacity and lead to structural distortions. Alternatively, these changes could represent an early stage of mitochondrial remodelling, involving the clearance of damaged mitochondria and the biogenesis of new mitochondria, with the observed dysmorphia reflecting immature or actively degrading organelles. This interpretation is supported by changes in gene expression that mirror these histological observations. The downregulation of Clk1 (Coq7), Nek5, and Gsdma in BS-treated kidneys is consistent with a reduction in pathways that are directly involved in mitochondrial maintenance and biogenesis. Clk1/Coq7 participates in coenzyme Q biosynthesis, Nek5 regulates mitochondrial DNA integrity, and Gsdma is associated with mitochondrial injury. Their suppression may therefore reflect an adaptive response to the presence of megamitochondria and altered mitochondrial dynamics after surgery. In parallel, the upregulation of genes such as Nlrp3, Fgr, Hck, Sult4a1, Rac2, Hvcn1, and Trem2 suggests the activation of signalling programmes related to oxidative stress, mitochondrial quality control, and immunometabolic adaptation. It is important to note that Rossi et al. [28] observed improvements in mitochondrial morphology over longer follow-up periods after bariatric surgery, suggesting that the initial disruption we observe is followed by a more sustained recovery of mitochondrial function.

As illustrated in Figure 6a, the global heatmap of sequenced genes revealed a tendency for BS and LFD samples to show mixed clustering, suggesting an overarching similarity in their transcriptomic profiles at a broad level. This observation is biologically significant, as it likely reflects shared fundamental physiological adaptations to weight loss itself. Both interventions successfully induce a degree of weight reduction and metabolic improvement, leading to a general convergence from the HFD-induced pathological state. The genes common to both groups in this global view represent these broad beneficial responses to weight loss that are shared. However, despite these global similarities, when comparing the transcriptomic results of both intervention groups, the bariatric surgery (BS) group exhibited a much more pronounced gene expression profile compared to the LFD and HFD groups. While the LFD group showed 30 and 16 overexpressed genes relative to the HFD and BS groups, respectively, the BS group displayed 143 and 105 overexpressed genes compared to the HFD and LFD groups, respectively. Remarkably, there were several genes that were commonly overexpressed in the BS group when compared with both the LFD and HFD groups (Supplementary Table S1). This pattern highlights a complex and specific renal response following bariatric surgery, characterized by two main functional clusters related to immune system activation: a pro-inflammatory and chemotactic response, and a regulatory signalling module. In this context, the integration of pathway-level information provides an added layer of biological interpretation that complements conventional gene expression analyses. Pathway activity-based approaches allow the identification of functional cellular states that are more robust to technical noise and biological variability than individual gene signals [12]. The regulatory cluster [35,36,37,38,39] comprises genes that modulate immune surveillance, T cell responses, cytokine signalling, and intracellular phagocytic pathways. This suggests a balance between inflammation and regulatory mechanisms that may promote tissue repair and functional recovery. Conversely, the pro-inflammatory cluster [40,41,42,43,44] comprises genes involved in leukocyte recruitment, inflammation amplification, tissue remodelling, and complement activation. Together, these findings indicate that bariatric surgery induces a complex renal immune response that could be key to reversing obesity-related renal damage and improving kidney function.

This study has several limitations that should be considered when interpreting the findings. First, only male Wistar rats were included, which precludes the assessment of potential sex-related differences in metabolic or renal responses to obesity and its treatment. Second, although diet-induced obesity is a well-established experimental model, it primarily reflects early stages of obesity-related glomerulopathy and cannot fully reproduce the complexity of human obesity and its associated comorbidities, which may limit the direct translation of these findings to clinical settings. Third, the relatively short follow-up period limits the ability to determine whether the mitochondrial changes observed in tubular cells after bariatric surgery represent a transient adaptive response or a sustained remodelling process. Fourth, transcriptomic analyses were performed on the bulk renal cortex, which integrates signals from multiple cell types and does not allow precise attribution of gene expression changes to specific compartments. More refined approaches, such as single-cell or spatial transcriptomics, could provide greater resolution. Finally, renal function was evaluated only through albuminuria; additional functional markers, including glomerular filtration rates or tubular injury biomarkers, would help clarify whether the structural recovery observed after bariatric surgery corresponds to a complete functional improvement. Despite these constraints, the consistent trends observed across histological, biochemical, and transcriptomic analyses lend support to the overall interpretation of the data.

Overall, these findings indicate that, in a pre-clinical rat model, bariatric surgery—unlike dietary restrictions—not only leads to effective weight loss and a complete histological reversal of ORG but also triggers significant mitochondrial restructuring in renal tubular cells. Notably, BS induces a unique renal immune signature involving both pro-inflammatory and regulatory pathways. This suggests that its renal benefits extend beyond weight reduction alone. The simultaneous restoration of glomerular morphology alongside signs of mitochondrial stress in tubular compartments points to a complex adaptive process. In this context, mitochondria appear to act as both targets and mediators of renal remodelling following bariatric surgery. This dual role highlights their potential importance in the post-surgical recovery of kidney function. Future research should determine whether these mitochondrial alterations represent a transient adaptive response or a sustained remodelling process with long-term implications for renal physiology and whether similar mechanisms occur in humans.

## 4. Materials and Methods

### 4.1. Establishment of the Obesity-Induced Animal Model

Twenty-eight Wistar Han male rats (300–350 g; Charles River Laboratories España S.A. (Barcelona, Spain), seven weeks old and not genetically modified, were housed and fed under standard diet conditions (SD) (TEKLAD 2014; % by weight: 14.3% protein, 4% fat, 18.0% NDF, 2.9 Kcal/g) for 2 weeks. Only male rats were used to avoid hormonal variability; this choice was approved by the institutional ethics committee. After that, animals were randomized into three main groups and fed with a high-fat diet (HFD) (TEKLAD 06414; % by weight: 23.5% protein, 34.4% fat, 27.3% carbohydrate, 5.1 Kcal/g) for 10 weeks when they became obese. Weight and food intake were measured weekly, and mice were considered to be obese when the weight gain was at least 25% of their baseline weight. After 10 weeks of HFD, 8 animals were immediately sacrificed (the HFD group), while the remaining rats entered the intervention phase, and 8 animals were fed a low-fat diet (the LFD group) (TEKLAD TD 94048; 12.4% protein, 4% fat, 68.3% carbohydrate, 3.6 Kcal/g) for 10 weeks while 12 animals underwent bariatric surgery (sleeve gastrectomy) (the BS group) and were fed an SD until the end of the study (Figure 1). Three out of twelve animals died after surgery. All diets were from Harlan Laboratories, Indianapolis, IN, USA. Rats were kept in a metabolic cage for the last 24 h prior to organ collection to ensure that the urine did not come in contact with feces or feed.

### 4.2. Bariatric Surgery

Bariatric surgery was performed under isoflurane anesthesia using a rodent surgical mask connected to a veterinary anesthesia machine. Prior to surgery, animals received a special liquid diet for two days and fasted for eight hours, with free access to water until two hours before the procedure. A 3.5 cm midline laparotomy was performed below the xiphoid process to gain access to the peritoneal cavity. The stomach was carefully identified, mobilized, and exteriorized. A crucial step involved the precise demarcation of the portion to be resected: a consistent gastric resection of approximately 70% of the total stomach volume, including the greater curvature and most of the fundus, was performed. This was achieved by creating a tubular gastric passage along the lesser curvature, from the esophagus to the duodenum, and ensuring that the resected portion corresponded to a distance of 1.5 cm. This resection was performed using a fine-tipped electrocautery device while meticulously ensuring hemostasis with bipolar electrocoagulation. The gastric remnant was then carefully inspected for any signs of bleeding or leakage. The technique, a vertical sleeve gastrectomy tailored for Wistar rats, rigorously adhered to established principles of human bariatric procedures [14], particularly concerning the preservation of the pylorus and vagal innervation.

After confirming hemostasis and integrity of the gastric staple line/suture, the abdominal cavity was closed in two layers: the muscle and peritoneum were closed with a 4-0 absorbable suture in a continuous pattern, and the skin was closed with a 4-0 non-absorbable suture. Post-operatively, animals received subcutaneous analgesia and were monitored daily for signs of pain, infection, or distress. They were reintroduced to a liquid diet for 2 days, followed by a soft diet for 3 days, and then switched back to a standard diet until the end of the study. Three out of twelve animals died after surgery, which is within the expected range for this procedure in a rodent model.

### 4.3. Tissue and Sample Collection and Assessment of Albuminuria

The kidneys were harvested from each animal and immediately stored in a saline solution at 4 °C to remove excess adipose tissue. A quarter of each renal sample was fixed in formaldehyde and embedded in paraffin for immunohistochemical and histological analyses; another quarter was frozen in an isopentane bath and preserved at −80 °C. The remaining tissue was immersed in RNA-later stabilization solution (Thermo Fisher Scientific Inc., Waltham, MA, USA) and stored at 4 °C for subsequent gene expression analyses (25 biopsies were used for transcriptomic and morphological studies). Urine samples from 25 rats were centrifuged at 2000× *g* for 10 min, and supernatants were aliquoted and stored at −80 °C for biochemical analysis.

Albuminuria was measured by using colourimetric enzyme-linked immunoassays (Abcam, Cambridge, UK) with a sensitivity of 0.44 ng/mL and a working range of 0.195–50 ng/mL. Ten microliters of urine, diluted to 1:100 with the appropriate buffer (including standards), were added to wells precoated and blocked with a rat-albumin-specific antibody. After incubation with a biotinylated anti-albumin detection antibody and streptavidin–peroxidase conjugate, color development was achieved using 3,3′,5,5′-tetramethylbenzidine as the substrate. All samples were processed in triplicate.

### 4.4. Morphometry, Histology, and Transmission Electron Microscopy (TEM)

Standard histopathology techniques were applied to 3 µm thick rat kidney sections. The glomeruli, particularly those sectioned through the hilum, were morphometrically analyzed for area and mesangial expansion. Thirty glomeruli per sample were assessed in periodic-acid–Schiff-stained and hematoxylin-and-eosin-stained sections. Slides were scanned at a 40 µm resolution using a PANNORAMIC^®^ 1000 Flash DX scanner (3DHISTECH Ltd., Budapest, Hungary) and analyzed using QuPath (v0.4.4). A mesangial matrix increase was defined as increased mesangial extracellular material with an interspatial width of >2 mesangial cell nuclei in one or more peripheral mesangial areas. Mesangial cell proliferation was defined as the presence of more than three mesangial cells surrounded by the mesangial matrix in an intact glomerular segment in 3 µm thick sections. Podocyte hypertrophy was defined as podocyte enlargement with large nuclei and prominent nucleoli, with or without intracytoplasmic protein resorption droplets.

TEM was used to analyze 3 rat samples (1 in the LFD group, 1 in the HFD group, and 1 in the BS group), as per the standard protocol. Tissues from the paraffin blocks were dewaxed, hydrated, post-fixed, dehydrated, and embedded in epoxy resin. Ultrathin sections were cut using a Leica Ultracut microtome and stained with uranyl acetate and lead citrate using a Leica EM stainer. Samples were examined using a JEM 1010 TEM (JEOL USA, Inc., Peabody, MA, USA) at 80 kV, and digital images were captured using an Orius CCD camera (Gatan Inc., Pleasanton, CA, USA).

### 4.5. Total RNA Isolation

RNA from 25 (8 HFD, 8 LFD, and 9 BS) rat kidneys was obtained from frozen tissue blocks using Trizol Reagent (Invitrogen, Thermo Fisher, Waltham, MA, USA). Genomic DNA was removed by incubation with DNase I for 10 min (Invitrogen), and QC was assessed in a TapeStation 2200 (Agilent, Santa Clara, CA, USA). Half of the RNA from the kidneys of rats was used for RNA-Seq experiments, and the rest was used for other purposes.

### 4.6. Library Preparation, RNA Sequencing, and Data Analysis

In total, 25 RNA-Seq libraries were prepared from 1 µg of the total RNA using an NEBNext^®^ Ultra™ II directional RNA library prep kit for Illumina^®^ (New England Biolabs, NEB, Ipswich, MA, USA). The HiSeq 2500 system generated 40–50 M reads/sample. FastQC was used to assess the read quality, and Cutadapt was used to remove adapters. The Salmon tool was used to map and quantify transcripts to the Rnor6.0 genome. DESeq2 v1.24.0 was used for differential expression analysis. The bioinformatics pipeline was executed on high-performance computing resources to efficiently manage the computational complexity of processing a comprehensive dataset comprising 25 RNA-Seq libraries (from 33 samples, totaling 132 raw fastq.gz files). This modular pipeline was designed for scalability, ensuring its adaptability for larger cohorts or deeper sequencing in future studies. Robustness was a key consideration throughout the analysis. Initial read quality was rigorously assessed using FastQC (v0.11.9) and MultiQC (v1.8), confirming acceptable quality scores and identifying potential issues like adapter contamination or sequence biases. Reads were then meticulously trimmed with Cutadapt (v2.10) to remove adapter sequences and low-quality bases (Phred score < 20), enhancing data integrity. Salmon (v1.3.0) was chosen for its robustness in accurate, bias-aware quantification of transcript expression, while DESeq2 (v1.24.0) performed differential expression analysis, inherently accounting for library size differences and data variability through its empirical Bayes shrinkage estimation. This comprehensive quality control and robust analytical framework was critical to ensure reliable and reproducible results.

### 4.7. Gene Ontology and Pathway Analyses

The global analysis of Biological Significance was applied according to Gene Ontology categories (considering three distinct aspects of how gene functions can be described: molecular function, cellular component, and biological process) and Reactome Pathway analysis. To further ensure the robustness of our interpretations, multiple testing correction was applied using the Benjamini–Hochberg method to control the False Discovery Rate (FDR). Additionally, an independent filtering step, implemented via DESeq2, was utilized to optimize statistical power by removing weakly expressed genes, thereby enhancing the reliability of the pathway enrichment results. Principal component analysis (PCA) was also performed on the normalized gene expression data to verify sample clustering and the absence of significant batch effects, contributing to the overall robustness of the dataset.

### 4.8. Statistical Analysis

Descriptive analyses summarized qualitative and quantitative characteristics as percentages and mean values plus standard errors (or median values and interquartile ranges), respectively. The main characteristics between the study and control groups were compared using an unpaired *t*-test or X^2^ test, as appropriate. The *t*-test was applied to non-parametric data after log transformation. For comparisons among experimental groups (HFD, LFD, and BS), a global statistical approach was applied. A one-way ANOVA was specifically used for albuminuria analysis. Fisher’s exact test for meta-analysis with Benjamini–Hochberg’s FDR correction was used to calculate the significant targeted biological processes and KEGG pathways. The complete linkage clustering method was used for the hierarchical clustering of pathways and miRNAs, with squared Euclidean distances as distance measures. Absolute *p*-values were used in all the calculations, considering the significance levels of the interaction. A two-tailed *p*-value of ≤0.05 denoted statistical significance. Statistical analyses were performed using SPSS (version 15.0; SPSS, Chicago, IL, USA).

## Data Availability

The original contributions presented in this study are included in the article/Supplementary Materials. Further inquiries can be directed to the corresponding author(s). The transcriptomic data supporting the findings of this study are available in the repository Gene Expression Omnibus (GEO) at https://www.ncbi.nlm.nih.gov/geo/ (accessed on 20 December 2025) (accession number: GSE314677).

## Supplementary Materials

The following supporting information can be downloaded at https://www.mdpi.com/article/10.3390/ijms27020839/s1.

## Abbreviations

The following abbreviations are used in this manuscript:

CKD	Chronic Kidney Disease
ORG	Obesity-Related Glomerulopathy
HFD	High-Fat Diet
LFD	Low-Fat Diet
BS	Bariatric Surgery
mGFR	Measured Glomerular Filtration Rate
SD	Standard Diet
NDF	Neutral Detergent Fibre
RNA	Ribonucleic Acid
miRNA	MicroRNA
RNA-Seq	RNA Sequencing
QC	Quality Control
DNA	Deoxyribonucleic Acid
DESeq2	Differential Expression Sequencing 2
GO	Gene Ontology
TEM	Transmission Electron Microscopy
FDR	False Discovery Rate
KEGG	Kyoto Encyclopedia of Genes and Genomes
SPSS	Statistical Package for the Social Sciences
3R	Replacement, Reduction, and Refinement
H&E	Hematoxylin and Eosin
PAS	Periodic Acid–Schiff
RNA-later	RNA Stabilization Solution
