# Supplementary material for: Bariatric Surgery Reverses ORG and Exhibits a Distinct Transcriptomic Profile Compared to Weight Loss Through a Low-Fat Diet
Source: Int J Mol Sci. 2026 Jan 14;27(2):839. doi: 10.3390/ijms27020839 (PMC12841304; doi:10.3390/ijms27020839)

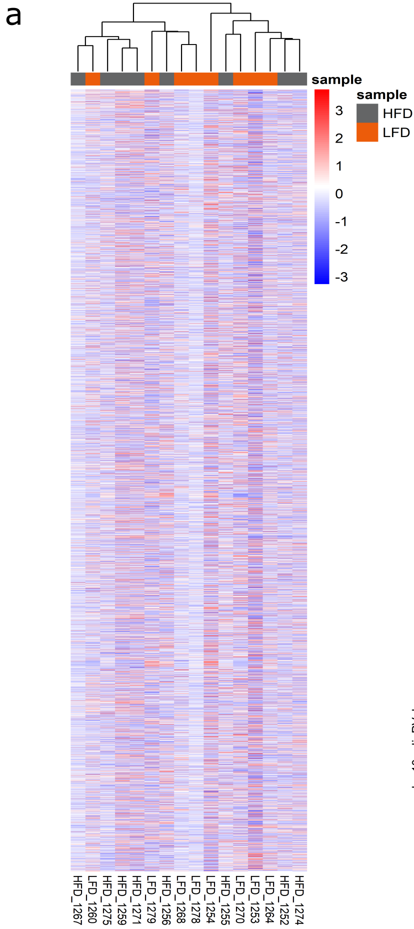

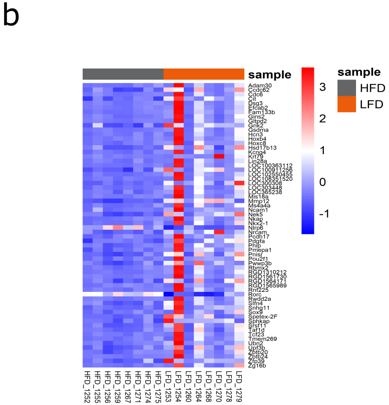

c

Gene symbol	log2FoldChange	adjusted p val
Mmp12	2,5032	0,0016
LOC100363112	2,0557	0,0130
RGD1561730	2,0465	0,0076
LOC300308	1,9344	0,0045
Spetex-2F	1,8176	0,0146
LOC108351520	1,7806	0,0079
Tcf23	1,7774	0,0116
Mis18a	1,7553	0,0159
Rnf225	1,7397	0,0145
Nrcam	1,7238	0,0116
Snhg11	1,6906	0,0098
Rwdd2a	1,6691	0,0125
Efcab2	1,6114	0,0090
Fam133b	1,5763	0,0028
Gltpd2	1,5729	0,0189
LOC102550455	1,5246	0,0045
Cdc6	1,4599	0,0168
Gsdma	1,3816	0,0053
Hoxb4	1,3521	0,0167
Nkap	1,2429	0,0168
Pmepa1	1,1946	0,0045
LOC303448	1,1940	0,0116
Cit	1,1579	0,0145
Kcng4	1,1536	0,0189
Phip	1,0635	0,0115
LOC100911256	1,0212	0,0177
Ccdc62	1,0208	0,0079
RGD1310212	0,9990	0,0180
Nek5	0,9982	0,0152
Pou2f1	0,9669	0,0167
Rorc	-0,8159	0,0052
Parp16	-0,5143	0,0125
Cry1	-0,5044	0,0286

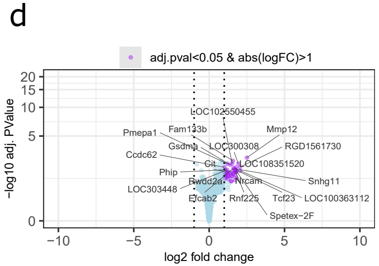

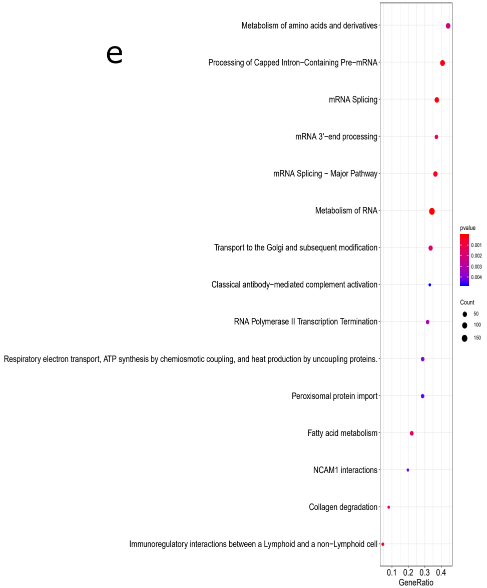

Supplement: Supplementary file 1 [file ijms-27-00839-s001.zip › Suppl 1.pdf]
