# Supplementary material for: Bariatric Surgery Reverses ORG and Exhibits a Distinct Transcriptomic Profile Compared to Weight Loss Through a Low-Fat Diet
Source: Int J Mol Sci. 2026 Jan 14;27(2):839. doi: 10.3390/ijms27020839 (PMC12841304; doi:10.3390/ijms27020839)
Supplement: Supplementary file 1 [file ijms-27-00839-s001.zip › Supplementary Table1.pdf]

Table 1. Genes Differentially Overexpressed in BS Compared to LFD and HFD

Biological Pathways Involved	Differentially Overexpressed Genes in BS
**Signaling by GPCR**	Adgre1, Ccl19, Ccl21, Pik3r5
**GPCR Downstream Signalling**	Adcy7, C3, Ccl19, Ccl21, Pik3r5
**GPCR Ligand Binding**	Adgre1, Ccl19
**Signaling by Rho GTPases**	Arhgap30, S100a8, S100a9, Rac2
**Adaptive Immune System (IS)**	Cd4, Itgal, Itgb2, RT1-Da, Siglec1
**Neutrophil Degranulation**	Cd53, Cd68, Mmp8, Nfam1, Tnfrsf1b
**Chemotaxis and Taxis**	Csf3r, Cx3cr1
**Innate Immune Response**	Cfi, Clec7a, Fgr
**Cytokine Signaling in IS**	Cd4, Hck, Il10ra, Il21r
**Antimicrobial Peptides**	Cd4, S100a8, S100a9
**Signaling by Interleukins**	Cd4, Hck, Il10ra, Il21r
**TCR Signaling**	Cd4, RT1-Da
**Hemostasis**	Itgal, Itgb2, Itgb7, Pik3r5, Rac2
**Extracellular Matrix Organization**	Itgal, Itgb2, Itgb7, Mmp8
**Integrin Cell Surface Interactions**	Itgal, Itgb2, Itgb7
**Regulation of Cell Activation**	Clec4d
**Regulation of Vesicle-Mediated Transport**	Fcgr2b, Fgr
**Wound Healing**	Clec10a
**Leukocyte Differentiation**	Clec4d
**Developmental Biology**	Trem2
**Metal Sequestration by Antimicrobial Proteins**	S100a8, S100a9
**Rhodopsin-like Receptors**	Ccl19
