# Supplementary figures and images for: Bariatric Surgery Reverses ORG and Exhibits a Distinct Transcriptomic Profile Compared to Weight Loss Through a Low-Fat Diet
Source: Int J Mol Sci. 2026 Jan 14;27(2):839. doi: 10.3390/ijms27020839 (PMC12841304; doi:10.3390/ijms27020839)

a. Biological processes

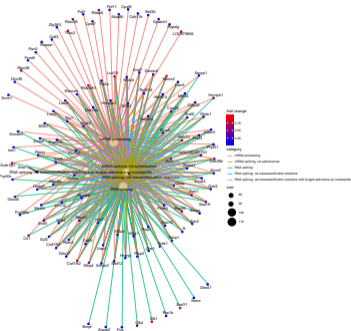

b. Cellular components

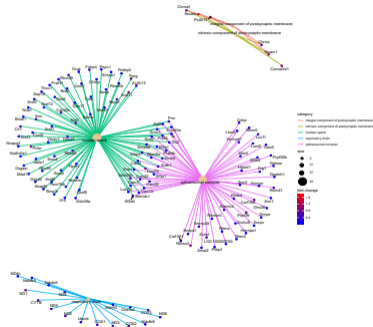

### c. Molecular functions

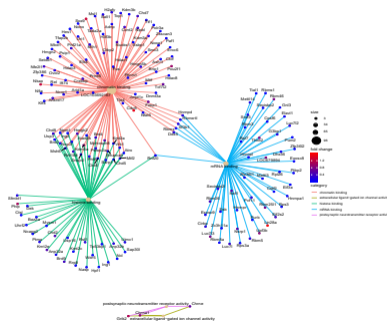

Supplement: Supplementary file 1 [file ijms-27-00839-s001.zip › Suppl 2.pdf]

a. Biological processes

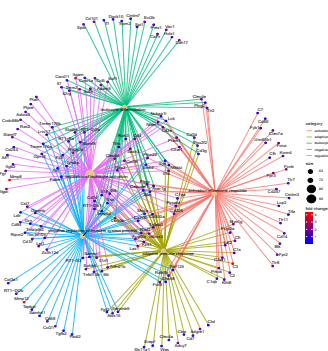

b. Cellular components

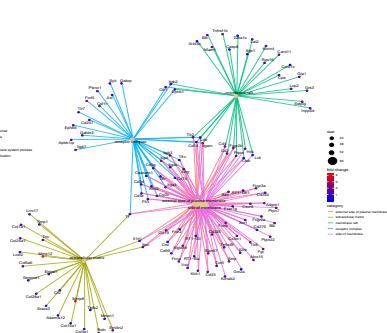

c. Molecular functions

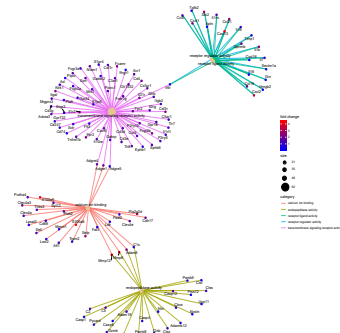

Supplement: Supplementary file 1 [file ijms-27-00839-s001.zip › Suppl 4.pdf]
