# Supplementary material for: Bariatric Surgery Reverses ORG and Exhibits a Distinct Transcriptomic Profile Compared to Weight Loss Through a Low-Fat Diet
Source: Int J Mol Sci. 2026 Jan 14;27(2):839. doi: 10.3390/ijms27020839 (PMC12841304; doi:10.3390/ijms27020839)

a

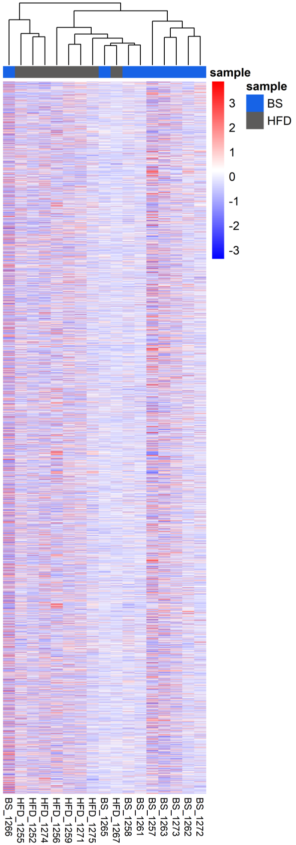

b

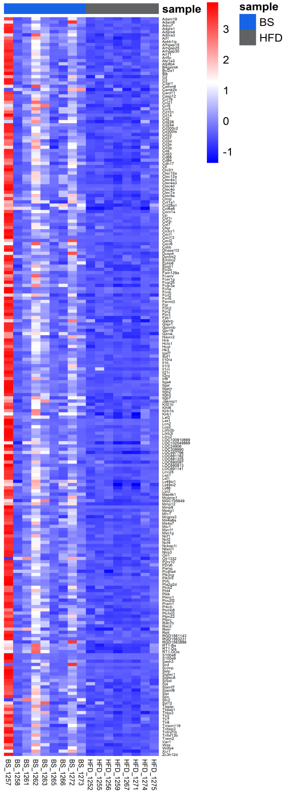

c

symbol	log2FoldChange	padj
S100a8	4,4252	5,26E-05
Cxcl13	4,3287	4,44E-12
Mmp8	4,2535	1,35E-04
Scd	4,2136	7,49E-03
Mmp12	4,2097	5,81E-14
C3	3,9394	4,22E-16
S100a9	3,8615	1,44E-04
Siglec1	3,3764	7,02E-05
Itgam	3,1728	7,67E-07
Pla2g2d	3,0896	8,51E-05
Pirb	2,9927	9,26E-03
Mcemp1	2,8571	4,25E-03
Stc2	2,7871	9,12E-05
Scimp	2,5701	1,66E-04
Sirpd	2,5594	4,69E-03
LOC24906	2,5390	3,56E-04
Hk3	2,4650	3,14E-04
Chi3l1	2,4113	3,56E-04
Lcn2	2,3823	1,20E-03
Ccl21	2,3663	9,12E-05
Csf3r	2,3493	8,69E-04
Siglec8	2,3426	4,29E-04
Selp	2,3358	4,62E-03
Itgal	2,3334	1,05E-05
Fcamr	2,3307	1,53E-04
Il1b	2,2803	1,05E-05
Fcgr2b	2,2561	1,54E-04
Mpeg1	2,2511	9,70E-06
Clec4d	2,2195	2,68E-03
Adam8	2,1629	2,68E-03
Trem2	2,1449	9,26E-03
Cd33	2,1300	8,27E-04
Myo1g	2,1277	2,96E-04
Adgre1	2,0243	4,29E-06
Fcrl2	1,9544	7,60E-03
LOC497796	1,9500	9,70E-06
Evi2b	1,9294	5,34E-04
Il21r	1,9243	1,36E-03
Nfam1	1,9167	1,03E-05
Cd4	1,9061	9,03E-06
Gpnmb	1,9011	2,48E-04
Clec4a3	1,8489	5,93E-05
Clec9a	1,8031	4,90E-04
Ccl19	1,7843	3,18E-03
Pou2f2	1,7682	4,43E-04
Azi2	-0,22933	0,016902
Nrbf2	-0,24306	0,008943
Timm29	-0,30406	0,016949
Cdip1	-0,37762	0,012625
Fbxo22	-0,39887	0,017239
Lonrf3	-0,65131	0,015978
Cry1	-0,65616	0,000885
Ahsa2	-0,74217	0,001808
Rorc	-0,75722	0,001680
S100g	-0,79072	0,016549
Pklr	-0,87748	0,015391

d

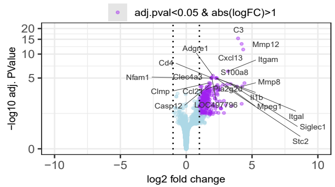

e

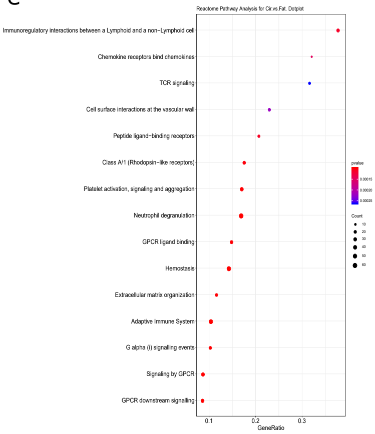

Supplement: Supplementary file 1 [file Suppl 3.pdf › Suppl 3.pdf]
